# Childhood migration experience and adult health: evidence from China’s rural migrants

**DOI:** 10.1186/s13690-024-01280-x

**Published:** 2024-04-22

**Authors:** Xiaohong Li, Shiyan Qiao, Dongying Zhang

**Affiliations:** College of Economics, Guizhou University, 550025 Guiyang, China

**Keywords:** Childhood migration, Adult health, Rural migrants, Migration distance, Gender difference

## Abstract

**Background:**

Place of residence plays an influential role in shaping individual development, and studies have established links between Childhood migration experience (CME) and health outcomes through maturity. Over the past three decades, China has undergone one of the largest rural-to-urban migrations, however, little is known about the effect of CME on rural migrants’ adult health in China.

**Methods:**

Data from 7035 members of the 2016 and 2018 China Labor-force Dynamics Survey were analyzed. CME was measured by whether the place of residence and place of birth changed at the age of 14 years. Three measures of health (self-assessed health, BMI, and mental health scale) were obtained. Causal inferential analysis was performed, using the Probit model, the OLS model and the Propensity Score Matching (PSM) method, to explore the impact of CME on the adult health of rural migrants.

**Results:**

Overall, compared to individuals who did not migrate in childhood, the probability of reporting “very unhealthy”, “rather unhealthy”, and “fair” in the self-assessed health of the rural migrants with CME decreased by 0.23%, 1.55%, and 5.53%, the probability of reporting “healthy” and “very healthy” increased by 1.94% and 5.38%, the probability of BMI within the normal range was higher by 7.32%, and the mental health test scores were 0.2591 points higher significantly. Furthermore, in comparison with childhood non-migration, both cross-county and cross-city migration promoted the health status of rural migrants, but the positive effect of cross-province migration was not significant; from the gender perspective, CME could more dramatically improve rural women’s adult health than men, especially in mental health.

**Conclusion:**

CME can significantly improve adult health, including physical and mental health, and the positive effect is more obvious among women, helping to reduce gender differences in health. For the migration distance, attention can be focused on the long-distance migrating individuals, who should get more support.

**Table:** 

Text box 1. Contributions to the literature	
•Previous studies have shown that childhood experiences are closely related to health, however, little literature has focused on CME and adult health, especially in developing countries. •Moving to opportunity in childhood significantly improved adult health, including physical and mental health. •Compared to short-distance migration, long-distance migration during childhood did not contribute significantly to the adult health of rural migrants. •CME could more dramatically improve rural women’s adult health than men’s, especially mental health. •These findings provide empirical support for “moving to opportunity” from developing countries.	

## Introduction

Moving to urban areas is a key way for rural residents to improve employment opportunities, incomes and living standards [1]. With the reform of China’s urban and rural household registration system, the cost of migration has been decreasing [2]. According to the latest data, China’s migrant population reached 375.8 million in 2020, a 69.73% increase compared to 2010 [3]. Family migration has become a new trend [4], and the number of migrant children has expanded to about 71.09 million in 2020 [5]. However, rural children may face major challenges in terms of nutritional health and need to adapt to changes in the local social environment. More empirical evidence, especially from developing countries, is still needed on the potential relationship between CME and the adult health of rural migrants.

Although diet and exercise directly influence physical health [6, 7], the accessibility of economic, social, and cultural resources also impacts health [8]. Therefore, it is crucial to go beyond the individual level and understand how family context and social resources influence health [9]. Weaver et al. [10] and Collyer [11] verified that economic, social, and cultural capital cause changes in dietary habits and exercise frequency by influencing lifestyle and resource allocation, thus improving individual health. For rural children, migration not only represents a change in the physical location, but may also alter different combinations of economic, social, and cultural capital during their life cycles, which may have far-reaching and long-lasting effects on their future health. Children’s development during childhood is irreplaceable for their lifelong health, nutrition and well-being, and plays a vital role in family happiness and social mobility. As an important part of the future labor pool, rural migrant children’s healthy human capital in adulthood is also closely related to the country’s development, so it is necessary to explore the impact of CME on their adult health. Moreover, the study scope of migration effects can be expanded from short-term to long-term, thereby offering valuable insights into migration effects within the context of the life cycle.

Extensive research has linked childhood experiences to adult health, but few studies have directly focused on CME in developing countries. Shonkoff et al. [12] noted that childhood environmental changes can influence an individual’s health across the lifespan. Further, childhood adversities, such as undernutrition, low social status, and lack of parental presence, increase the risk of chronic disease in adulthood [13, 14]. Empirical evidence from developed countries nearly demonstrates the negative impact of CME on adult health. Webb et al. [15], based on a national sample from Danish, found that migration experiences before the age of 15 could increase health risks in midlife, including suicidal behaviors, psychiatric disorders, and natural death. Using cohort data from the United States, Dong et al. [16] and Alvarado [17] also stated that CME was strongly associated with depression, smoking, alcohol abuse, and obesity in adulthood. Additionally, Simsek et al. [18] concluded that CME is more likely to have detrimental effects on health by conducting a meta-analysis of 90 studies published between 1989 and 2020.

Migration in developed countries is mostly due to bankruptcy, unemployment, etc., and such involuntary migration can be a stress source for children [19], ultimately negatively impacting health. However, China’s rural migration is unique because the Chinese pattern is mainly rural-urban migration, which is the “moving to opportunity”. The “dual structure model” [20], the “push-pull theory” [21] and the “rural-urban labor migration model” [22] all provided the theoretical basis for explaining “moving to opportunity” in developing countries. In China, migrant workers earn more than non-migrants, with an income gap as high as 29.27% in 2021 (Migrant Worker Monitoring Survey Report, 2012–2022). Duan et al. [23] also found that compared with non-migrated rural residents, the welfare of rural residents who migrated to cities in 2000–2010 and 2010–2017 improved by 37.93% and 29.75%. Thus, the migration of rural migrants in China is usually a rational choice to maximize utility. With reference to existing studies [24, 25], and combining the existing information in the data, this paper defines the people whose place of birth and residence changed at age 14 as the samples with CME. So, as the largest developing country, what is the impact of the CME of China’s rural migrants on adult health? Does this effect change with migration distance? Additionally, in the context of rural-urban migration, where a marked preference for sons over daughters in rural areas is being challenged, does CME result in gender differences in health outcomes?

To respond to the above questions, data from the 2016 and 2018 China Labor-force Dynamics Survey were used to explore the impact of CME on adult health. Compared with the existing studies, the marginal contributions of this paper are as follows. Firstly, using the life course as an entry point, the study focuses on childhood migration experiences, an important factor that has been neglected in most of the studies on the health of migrant populations, and extends the studies on migrant children from static analysis to dynamic analysis, especially providing empirical evidence from China that “moving to opportunity” affects adult health. Secondly, analyzing gender differences in health dimensions from an individual microscopic perspective, as well as migration distance heterogeneity, and the relevant findings may bring insights for promoting migrant health. Moreover, the PSM method, the construction of Ratio index and the exclusion of relevant variables are used to mitigate endogeneity, which make the estimates more reliable.

The rest of this paper is structured as follows. The second part shows the research hypothesis based on the theoretical analysis. The third part describes data sources, selected variables and model construction, followed by empirical analysis in the fourth part. Finally, the paper ends with some concluding remarks and discussions.

## Theoretical analysis and research hypotheses

### Direct effects of CME on adult health of rural migrants

For exploring the relationship between individuals’ childhood experiences and adult health, the “life course theory” usually has strong interpretive power. The theory views the life course as a sequence of multiple life events on a temporal axis, emphasizing that the same set of life events, when ordered differently in time, can have different effects on individuals [26]. Currently, studies have been conducted to validate this theory further. One explanation is the “sensitive period model”, which suggests that experiences during sensitive periods (embryonic, childhood, etc.) have lasting and potentially irreversible biological effects, ultimately influencing health across the lifespan [27, 28]. Another explanation is the “adolescent pathway model”, meaning that childhood experiences expose people to different social conditions, which puts pressure on health [29] and have far-reaching cumulative effects over time [30].

Although the above studies provide theoretical references, there is no direct evidence of a possible relationship between CME and adult health in China. CME implies a geographic change from rural to urban, and affects individuals’ development by changing the resources and opportunities available. Notably, migration motivation has different impacts on the availability of economic, social and cultural resources [18], so it is crucial to analyze the migration motivation in the Chinese social context.

According to the new economics of labor migration [31], individuals not only consider individual utility maximization when making migration decisions but highly value family benefits. In China, most rural migration is intended for better economic, social and cultural resources [4], that is, moving to opportunity. Rural children’s migration is the result of rational choices made by the paternal generation [32]. In the long run, the short-term disadvantages of migration are offset by improved family income, social capital, and cultural attitudes [33]. Therefore, rural migrant children can have more opportunities and resources, which has a cumulative effect on adulthood and may positively impact their health. Specific analyses are presented below:

From the family level, the family structure of migrant children is relatively complete [32], the importance of which has been verified for children’s healthy growth. On the one hand, due to their parents’ higher income and improved dietary structure, migrant children have a solid health foundation [34, 35]. On the other hand, parental care and companionship can guide children to form healthy behavioral habits and provide sufficient emotional support, which improves children’s physical and mental health [36, 37], and the positive effects are transmitted to adulthood, thus enhancing the adult health of rural migrant children.

From the perspective of migrant children, CME may deteriorate mental health in the short term by disrupting their previous social capital and learning continuity. However, it should not be ignored that children are inherently resistant to adversity. After migrating to cities, rural children’s social networks outside of the countryside begin to expand [38], and high-quality urban social capital contributes to increasing their resilience [39], in the long run, which can counterbalance the negative impact of CME. Then, family usually plays a more critical role than school in determining academic achievement [40]. For example, parents provide guarantees in life care, learning counselling, and role modelling to enhance rural migrant children’s self-efficacy and reduce mental health risks [41, 42]. In addition, better urban surroundings provide a “modeling effect” for rural migrant children, and growing up in a community where peers and adults value health leaves a deep imprint on them, thus motivating them to maintain a healthy weight [17]. Hence, hypothesis 1 is put forward.

#### H1

CME is conducive to improving the adult health of rural migrants.

### Heterogeneous effects of CME on adult health of rural migrants

Distance plays a pivotal role when individuals find a utility-maximizing location by weighing the costs and benefits of migration [4]. Generally, the farther the migration distance, the greater the economic benefits of migration, but as the distance increases, the greater macroscopic changes in politics, economy and society and microscopic changes in lifestyles and values. At this time, migrated individuals face unfamiliar social networks and social environments, leading to difficulties in adaptation, which may cause some stress on health in the short term [25, 43], then further transmitted to the health performance of adulthood. Therefore, hypothesis 2 is put forward.

#### H2

Compared to short-distance migration, long-distance migration during childhood does not contribute significantly to the adult health of rural migrants.

After migration, parents’ parenting attitudes change, creating differences in the investment in their sons’ and daughters’ health capital. In China’s rural areas, the long-standing concept of “raising children to support the elderly” leads to a preference for boys over girls [44, 45]. In general, women suffer disadvantages in most health indicators [46]. Fortunately, with rapid urban industrialization, CME may weaken gender differences in health dimensions. On the one hand, after migrating from rural to urban areas, the fostering concepts of migrating parents gradually converge with those of urban residents [47]. This is conducive to strengthening migrating parents’ cognitive practice of “gender equality”, thus reducing gender differences in the health investment. On the other hand, girls outperform boys in language proficiency and memory tests [48], whose linguistic and cultural capital advantages enable them to adapt to new environments more quickly, so female migrants may have better health performance in adulthood [49, 50]. Based on this, hypothesis 3 is proposed.

#### H3

CME can significantly improve adult health in women more than in men.

**Fig. 1:**
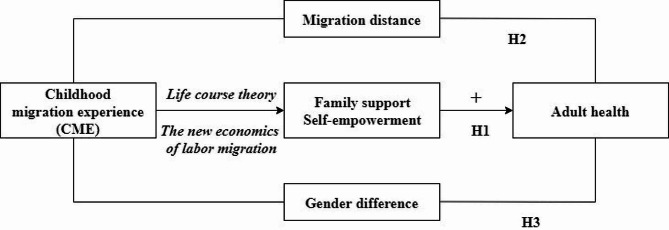
The theoretical framework

The theoretical framework diagram is shown in Fig. 1.

## Materials and methods

### Source of data

The study used data from the China Labor-force Dynamics Survey (CLDS) implemented by the Center for Social Science Survey of Sun Yat-sen University in 2016 and 2018. The survey covers 29 provincial-level administrative regions in China (except Hong Kong, Macao, Taiwan, Tibet, and Hainan), including information on migration, education, health and occupation of the labor force aged 15–64. In terms of the sampling methodology, CLDS adopts a multi-stage, multi-stratified, proportional to the number of laborers sampling method, and then utilizes the tracking survey method of rotating samples, ensuring the representativeness of the survey. Considering that this paper studies the rural migrants, only respondents who were in rural households from birth to age 14, born after 1970 (Those born after 1970 reached school age after the reform and opening up of China in 1978, and large-scale population movements began in China’s rural areas), and not in school were selected. In this paper, samples with missing variables were eliminated, and after cleaning the database, 7035 samples of data were finally obtained. Among them, the sample with CME is 569, and the sample without CME is 6466.

Moreover, it should be noted that despite the large number of rural migrants in China, the data limitations, as well as the limitations of this paper in terms of age at birth, household registration, migration status at age 14, and educational status, result in a small sample with CME. However, this does not affect the representativeness of the samples, which is similar to existing studies [4, 25, 47], and also meets the data needs for empirical analysis.

### Variables selection

#### Explained variable

Self-assessed health is a comprehensive assessment that reflects the states of the human body and mind, with good reliability, which has been widely used [51, 52]. It reflects the perception of objective physical health status. Illness, limited life ability, physical disability, etc., will lead to more negative self-evaluation of health [53]. On the other hand, as an integrated cognition, self-assessed health also permeates the influence of psychological status. People with positive attitudes are more likely to remain optimistic about their own health, thus making a higher level of health self-assessment [54]. Self-ratings of health may be modified by age or culture, but still be an effective measure of health status [55]. Therefore, this paper used the self-assessed health indicator as a proxy variable for overall health status. In conjunction with the CLDS questionnaire, the question “What do you think of your current health status?” was assigned the following values: very healthy = 5, healthy = 4, fair = 3, rather unhealthy = 2, and very unhealthy = 1.

However, although self-assessed health contains physical and mental health information, it is a little subjective and may introduce some deviation [56, 57]. In this regard, with reference to existing studies [46, 58, 59], this paper introduces the Body Mass Index (BMI) and the Mental Health Test to reflect physical and mental health, enhancing the reliability of the empirical test further. The details are as follows: for BMI, this paper sets a binary variable based on WHO’s standard, and when BMI is too high ($$ \ge 25$$) or too low ($$ <18.5$$), physical health is assigned a value of 0; BMI is within the normal range ($$ 18.5 \le BMI <25$$), and the value is taken as 1. For mental health, the frequency of negative emotions in the last week was indicated, which included six aspects: feeling depressed, having a hard time doing anything, having a hard time sleeping, feeling lonely, crying, and giving up life. For example, the times of “feeling depressed” appeared in the past week ranged from 5 to 7 days, 3–4 days, 1–2 days, to less than 1 day, with a value of 0–3, and the scores of the above six questions were summed up. The scores for the above six questions were totaled, with higher scores indicating better mental health.

#### Explanatory variable

The period of childhood is defined as the time span from birth to 14 years old, and this classification is supported by the following reasons. The United Nations General Assembly defines “youth” as a group of people between the ages of 15 and 24 years old, so the period of 14–15 years old is usually an essential milestone in childhood. However, the CLDS only asked about changes of residence at age 14, and existing research mostly used the time node of 14 years old [15, 24, 25]. Hence, this paper determines whether an individual migrated or not through the retrospective question “Is the place of residence at the age of 14 the same as the place of birth” (accurate to the county), including not only movement at the age of 14 years, but movement before the age of 14 years which continues until the age of 14 years. If the answer is “no”, it means that the individual migrated in childhood, which takes the value of 1; otherwise, the value is 0. For migration distance, this paper compares the birthplace and residence of rural migrants at the age of 14, and categorizes the migration distance into childhood non-migration, cross-county migration (in the same city), cross-city migration (in the same province), and cross-province migration (in the same country).

#### Control variables

Following a wide range of literature [4, 47, 51, 52], the following control variables were selected: (1) individual characteristics, including gender, age, age-squared, marital status, education, health insurance, and lifestyle (smoking, drinking and exercise); (2) family characteristics, including parents’ education, parents’ marital status at age 14, and the number of siblings; (3) regional characteristics, with regional variables constructed at the provincial level based on place of birth. In particular, it is necessary to point out that the family characteristics variables and the regional variables do not vary with time trends, which can effectively mitigate the endogeneity of the study.

Descriptive statistics of the variables are shown in Table 1. To observe more visually the relationship between CME and health, a preliminary analysis of differences between groups was done, and the results are shown in Table 2. Consistent with the previous theoretical analysis, individuals with the CME were in better health; individuals with long-distance migration were in worse health than individuals with short-distance migration. Besides, males were significantly better than females in the self-assessed health and mental health dimensions, confirming existing research [46], and it remains to be seen whether the CME can improve gender differences in health. However, in terms of physical health measured by the BMI, women were better than men, which is closely related to China’s national conditions, where the allocation of family resources favors men over women, so men are prone to over-nutrition, resulting in excessive obesity. The above analyses have not considered the effects of control variables, so this paper conducts further empirical analyses.

**Table 1:** Synopsis of the variable description (*n* = 7035)

Variables	Code	Definition	Mean	Std. Dev
		**Explained variable**		
Self-assessed health	health	Very unhealthy = 1; rather unhealthy = 2; fair = 3; healthy = 4; very healthy = 5	3.8023	0.7991
Physical health	physical	$18.5\le \text{B}\text{M}\text{I}<25$, physical health = 1; otherwise, physical health = 0	0.6141	0.4868
Mental health	mental	Mental health test (0–18 points)	15.4533	2.6290
		**Explanatory variable**		
Childhood migration experience	migration	Whether migration occurred at age 14: Yes = 1; No = 0	0.0809	0.2727
		**Individual characteristics**		
Gender	female	Male = 0; Female = 1	0.5818	0.4933
Age	age	Actual age in the year of the survey (years)	35.5080	9.0529
Education	edu	Years of education (years)	9.2489	3.8918
Marital status	marry	In marriage = 1; otherwise, the value = 0	0.8152	0.3882
Health insurance	insur	Whether enrolled in health insurance: Yes = 1; No = 0	0.9035	0.2953
Smoke	smoke	Whether smoked daily ($\ge 1$cigarette per day, continuously for 1 year or more): Yes = 1; No = 0	0.2124	0.4090
Drink	drink	Drinking alcohol (at least 1 time per week): Yes = 1; No = 0	0.1674	0.3734
Exercise	exercise	Regular exercise in the last month: Yes = 1; No = 0	0.3006	0.4586
		**Family characteristics**		
Father’s education	fathedu	Years of education (years)	6.2220	3.7505
Mother’s education	mothedu	Years of education (years)	4.2451	3.7794
Parents’ marital status at age 14	pamarry	In marriage = 1; otherwise, the value = 0	0.9723	0.1642
Number of siblings	sibling	The number of siblings (number)	2.5048	1.7267

Note: There are 29 provincial administrative units in the birthplace, which are not shown here due to space

**Table 2:** Differences in the health between groups

	Self-assessed health		Physical health		Mental health	
	Mean (SD)	*P*	Mean (SD)	*P*	Mean (SD)	*P*
**Childhood migration**						
Yes (*n* = 569)	3.9613 (0.8320)	0.0000***	0.6731 (0.4695)	0.0019***	15.6591 (2.8495)	0.0710*
No (*n* = 6466)	3.7883 (0.7947)		0.6089 (0.4880)		15.4352 (2.6081)	
**Migration distance**						
**Control group**: no migration (*n* = 6466)						
cross-county migration in the same city (*n* = 419)	3.9356 (0.8794)	0.0009***	0.6850 (0.4651)	0.0013***	15.5895 (2.9749)	0.3007
cross-city migration in the same province (*n* = 75)	4.1066 (0.6057)	0.0000***	0.6400 (0.4832)	0.5809	16.0400 (2.3100)	0.0273**
cross-province migration in the same country (*n* = 75)	3.9600 (0.7433)	0.0504*	0.6400 (0.4832)	0.5809	15.6667 (2.6115)	0.4477
**Gender**						
Male (*n* = 2942)	3.9534 (0.7280)	0.0000***	0.5908 (0.4918)	0.0007***	15.9112 (2.4531)	0.0000***
Female (*n* = 4093)	3.6936 (0.8298)		0.6308 (0.4826)		15.1241 (2.7012)	

Note: * *p* < 0.1, ** *p* < 0.05, *** *p* < 0.01

### Datum model setting

In this paper, self-assessed health is used to reflect overall health, and the credibility of the outcome variable is enhanced by the BMI and mental health test, portraying the health status from the above 3 aspects. Since self-assessed health is an ordered categorical variable, the Ordered Probit model is used, and the model is set as follows:

1 $$ {health}_{i}^{*}={\alpha }_{0}+{\alpha }_{1}{migration}_{i}+{\alpha }_{2}{X}_{i}{+\epsilon }_{i}$$

Here, $$ {migration}_{i}$$ is an explanatory variable indicating whether the $$ {i}_{th}$$ sample migrated in childhood; $$ {X}_{i} $$denotes a series of control variables (including individual, family, and regional characteristics), $$ {\epsilon }_{i}$$ means the random error term, $$ {\alpha }_{0}$$ is the intercept term, and $$ {\alpha }_{1}$$ and $$ {\alpha }_{2}$$ are the parameters to be estimated. Besides, $$ {health}_{i}^{*}$$ is the potential self-assessed health status, which is related to the observable ordered series $$ {health}_{i}$$ as follows:

2 $$ { health}_{i}=\left\{\begin{array}{c}1, if {health}_{i}^{*}\le {r}_{0}\\ 2, if {{r}_{0}<health}_{i}^{*}\le {r}_{1}\\ 3, if {{r}_{1}<health}_{i}^{*}\le {r}_{2}\\ 4, if {{r}_{2}<health}_{i}^{*}\le {r}_{3}\\ 5, if {{r}_{3}<health}_{i}^{*}\end{array}\right.$$

Here, $$ {r}_{0}$$、$$ {r}_{1}$$、$$ {r}_{2}$$、$$ {r}_{3}$$ are cut points, indicating the parameters to be estimated. When $$ {health}_{i}^{*}\le {r}_{0}$$, respondents rated themselves as very unhealthy; when $$ {{r}_{0}<health}_{i}^{*}\le {r}_{1}$$, respondents rated themselves as rather unhealthy; and similarly for the remaining three cases.

Secondly, BMI is a binary discrete variable, so the Probit model is used, which is set as below:

3 $$ prob\left(physical=1\right)={\varnothing (\beta }_{0}{+\beta }_{1}{migration}_{i}{+\beta }_{2}{X}_{i}+{\mu }_{i})$$

Here, ${\mu }_{i}$ represents the random error term, ${\beta }_{0}$ is the intercept term, and ${\beta }_{1}$ and ${\beta }_{2}$are the parameters to be estimated. Finally, the individual’s mental health status is a continuous variable, so the OLS model is set as follows:

4 $$ {mental}_{i}={\vartheta }_{0}+{\vartheta }_{1}{migration}_{i}+{\vartheta }_{2}{X}_{i}{+\phi }_{i}$$

Where $$ {mental}_{i}$$ means the individual’s mental health status, $$ {\phi }_{i}$$ refers to the random error term, $$ {\vartheta }_{0}$$ is the intercept term, and $$ {\vartheta }_{1}$$ and $$ {\vartheta }_{2}$$ are the parameters to be estimated.

## Results and analysis

### Baseline regression analysis

Table 3 presents the results of CME on the adult health of rural migrants. Column (1) of Table 3 showed that CME had a significant positive effect on overall health (self-assessed health). From column (2) of Table 3, the impact coefficient of CME on physical health was 0.1959, which still showed a significant positive effect. As shown in column 3 of Table 3, the mental health test scores of rural individuals with CME were 0.2591 points higher than those who did not migrate in childhood, significantly at the 5% level. In summary, CME does improve the adult health status of rural migrants, confirming hypothesis 1 of the theoretical analysis, and providing direct empirical evidence that “moving to opportunity” can positively contribute to individual health. Moreover, the control variables are not the core of exploration, so we do not extend the control variable results.

Additionally, in columns (1) and (2) of Table 3, the estimated coefficients from the Probit model reflect only the direction and significance of the effect. For ease of interpretation, it is necessary to estimate the marginal effect of CME on overall health and physical health. As illustrated in column (1) of Table 4, compared with individuals who did not migrate in childhood, the probability of reporting “very unhealthy”, “rather unhealthy”, and “fair” in the self-assessed health of the rural migrants with CME decreased by 0.23%, 1.55%, and 5.53%, and the probability of reporting “healthy” and “very healthy” increased by 1.94% and 5.38%, and all of them were significant at the 1% level. Column (2) of Table 4 also indicated that the probability of BMI within the normal range was significantly higher by 7.32%. The above results for marginal effects are consistent with the baseline regression analysis in Table 3, again validating hypothesis 1.

**Table 3:** Baseline regression results

Variables	(1) Self-assessed health	(2) Physical health	(3) Mental health
migration	0.2150*** (0.0525)	0.1959*** (0.0582)	0.2591** (0.1235)
female	-0.2989*** (0.0342)	0.1055*** (0.0407)	-0.8080*** (0.0814)
age	-0.0267** (0.0125)	0.0917*** (0.0149)	-0.0398 (0.0300)
${\text{a}\text{g}\text{e}}^{2}$	0.0001 (0.0002)	-0.0014*** (0.0002)	0.0006 (0.0004)
edu	0.0161*** (0.0043)	0.0086* (0.0050)	0.0382*** (0.0107)
marry	0.0535 (0.0466)	-0.0547 (0.0563)	0.4364*** (0.1246)
insur	-0.0496 (0.0460)	-0.1468*** (0.0533)	0.1844 (0.1177)
smoke	0.0265 (0.0398)	0.0104 (0.0477)	-0.0530 (0.0932)
drink	0.0369 (0.0392)	-0.0058 (0.0467)	-0.2269** (0.0903)
exercise	0.1078*** (0.0303)	-0.0484 (0.0351)	0.1331** (0.0658)
fathedu	-0.0075* (0.0043)	-0.0072 (0.0050)	0.0340*** (0.0105)
mothedu	0.0105** (0.0044)	-0.0053 (0.0052)	-0.0099 (0.0102)
pamarry	0.0506 (0.0831)	-0.1448 (0.0974)	0.1166 (0.1916)
sibling	-0.0169* (0.0091)	-0.0027 (0.0106)	-0.1373*** (0.0226)
Constant term			16.2617*** (0.6027)
Region	√	√	√
N	7035	7035	7035
$$ {R}^{2}$$	0.0550	0.0214	0.0598

Note: Robust standard errors in parentheses. * *p* < 0.1, ** *p* < 0.05, *** *p* < 0.01. The same as tables below (Excluding Table 5)

**Table 4:** Marginal effects of childhood migration experience on self-assessed health and physical health

Variables	(1)	Variables	(2)
health = 1 (Very unhealthy)	-0.0023*** (0.0007)	physical = 0 (Unhealthy physical)	-0.0732*** (0.0217)
health = 2 (Rather unhealthy)	-0.0155*** (0.0038)	physical = 1 (Healthy physical)	0.0732*** (0.0217)
health = 3 (Fair)	-0.0553*** (0.0135)		
health = 4 (Healthy)	0.0194*** (0.0048)		
health = 5 (Very healthy)	0.0538*** (0.0131)		

### Robustness test

#### Estimations based on the propensity score matching method

Due to the “healthy migration effect” [60], a mixed regression of childhood migrants and childhood non-migrants may not satisfy random sampling. To minimize sample self-selection bias, this paper constructs a “counterfactual frame” through the PSM method to isolate the net effect of CME. Concretely, the study sample was divided into a childhood migration treatment group and a non-migration control group, and given the covariates, a “propensity score” was used to represent the conditional probability that an individual would enter the “treatment group”, and then individuals with similar characteristics but different CME were matched, whereby the difference in the health of the two groups could be considered as the net effect of CME.

In this paper, three methods of near-neighbor matching (1:4), caliper matching (*R* = 0.01), and kernel matching were used. Before measuring the ATT values, we conducted covariate balance tests and common support hypothesis tests, which were in line with expectations (due to space limitations, the results are not reported in the paper). As shown in Table 5, it is evident that the ATT values calculated by the three matching methods were all negative, with a slight difference in significance level, which is consistent with the results of the baseline regression analysis.

**Table 5:** ATT values for different matching methods

	(1) Self-assessed health	(2) Physical health	(3) Mental health
Near-neighbor matching (1:4)	0.1568*** (0.0496)	0.0796*** (0.0291)	0.2131 (0.1720)
Caliper matching (*R* = 0.01)	0.1450*** (0.0438)	0.0764*** (0.0226)	0.2626** (0.1325)
Kernel matching	0.1542*** (0.0318)	0.0724*** (0.0232)	0.2273** (0.1148)
Individual character	√	√	√
Family character	√	√	√
Region	√	√	√
N	7035	7035	7035

Note: Standard errors in parentheses, obtained with 50 iterations using the self-help method

#### Excluding the effects of omitted variable bias

PSM mainly alleviates the sample self-selection problem, and to minimize the estimation bias caused by omitted variables, this paper refers to related studies [43, 61], and adopts observable variables to discriminate the bias caused by unobservable variables. Specifically, the regression using two differentiated control sets constructs the following indices:

5 $$ Ratio=\left|\frac{{\widehat{\beta }}_{2}}{{\widehat{\beta }}_{2}-{\widehat{\beta }}_{1}}\right|$$

Here, $$ {\widehat{\beta }}_{2}$$ represents the estimated coefficient of the explanatory variable under all possible control sets, and $$ {\widehat{\beta }}_{1}$$ reflects the coefficient under a limited set of control variables. If the value of Ratio is larger, it means that the selected observable variables are more explanatory and the possibility of omitting unobserved variables is smaller. When $$ Ratio>1$$, it can be assumed that omitting variables does not have greater explanatory power for the estimation results compared to the selected observable variables, indicating the effect of omission bias interference is negligible. Given the complexity of the coefficients in the Probit model, in this part of the paper, the explained variables are all regarded as continuous variables, and the coefficients are estimated by using the OLS model [43].

We checked for omitted variable bias in the baseline regression. First, we constructed three sets: set 1 includes only the explanatory variable (CME); set 2 includes the explanatory variable and individual variables; set 3 includes all control variables, and the results are shown in Table 6. Regardless of how the explained variables were replaced, the Ratio values computed from sets 1 and 3, and sets 2 and 3 were much larger than 1. This means that if the omitted variables interfere with the estimation results, their explanatory power is at least 5.1821 times that of the selected variables. The baseline regressions have controlled for individual, family and regional variables as much as possible, so it is credible that omitted variables are less likely to interfere with the regression results.

**Table 6:** Tests for omitted variables

Explained variable: self-assessed health	Impact coefficient	Ratio
Set 1	0.1731	sets 1 and 3: 5.1821 sets 2 and 3: 6.3362
Set 2	0.1680	
Set 3	0.1451	
Explained variable: physical health	Impact coefficient	Ratio
Set 1	0.0642	sets 1 and 3: 8.2135 sets 2 and 3:7.4592
Set 2	0.0633	
Set 3	0.0731	
Explained variable: mental health	Impact coefficient	Ratio
Set 1	0.2239	sets 1 and 3: 7.3608 sets 2 and 3: 95.9630
Set 2	0.2564	
Set 3	0.2591	

Note: Set 1 (the explanatory variable); Set 2 (the explanatory variable + individual variables); Set 3 (the explanatory variable + all control variables)

#### Exclusion of relevant variables

CME impacts significantly adult health, however, migration after age 14 may also affect health [17, 25]. In the baseline regression, this paper distinguished the treatment and control groups by “whether the individual migrated at age 14”, but some of the samples also migrated after age 14, which may interfere with the research results. In response, this paper removed the samples with migration experience after age 14 for robustness testing, and the results were exhibited in Table 7. It is easy to see that CME helps to improve the health of rural migrants and passes the significance test, validating the robustness of the baseline regression.

**Table 7:** Deletion of the samples with migration experience after age 14

Variables	(1) Self-assessed health	(2) Physical health	(3) Mental health
migration	0.2441*** (0.0612)	0.2131*** (0.0662)	0.3173** (0.1437)
Constant term			16.4509*** (0.6766)
Individual character	√	√	√
Family character	√	√	√
Region	√	√	√
N	5266	5266	5266
$$ {R}^{2}$$	0.0604	0.0237	0.0630

### Heterogeneity analysis

The previous analysis focuses on hypothesis 1, which reflects the average effect of CME on the adult health of rural migrants. Theoretical analysis also shows migration distance differences and gender differences in this effect. Therefore, this paper further examines the heterogeneity of the impact of CME on adult health based on gender and migration distance, and the results are shown in Tables 8 and 9.

Given that long-distance migrants are likely to come from poorer places and have lower health capital before migration, we included the birthplace variable to mitigate this bias as much as possible, and the regression results are shown in Table 8. Compared with childhood non-migration, cross-county migration and cross-city migration could significantly improve adult overall health, but the positive effect of cross-province migration did not pass the significance test; in terms of physical health, only cross-county migration significantly promoted physical health; in terms of mental health, only cross-city migration had a significant positive effect. The results verify hypothesis 2, that is, the positive effect of long-distance migration on adult health is not significant compared to short-distance migration. Therefore, the family migration should consider the economic benefits and the integration costs to make the optimal decision, thus promoting the maximum welfare of the offspring.

Table 9 reported gender differences in the impact of CME on adult health. CME improved women’s overall health more than men’s and passed a seemingly unrelated estimation test (SUEST) at the 10% level, supporting hypothesis 3. Combined with Table 2, we could know that men were dominant in both self-assessed health and mental health, and fortunately, childhood migration was more favorable to women’s health. Further, this difference in overall health was not physical but mental health, meaning CME promoted women’s mental health more than men’s. The reasons are as follows: as a result of the enhanced cognitive practice of “equality between men and women” by parents who migrate to urban areas, rural girls can obtain more resource inputs after migration, and the advantage of language also enables them to adapt to the new environment more quickly, resulting in a more remarkable improvement in their physical and mental health; however, after migration, the increment of boys’ resource is too common to create a strong mental contrast with the pre-migration period, and only improves physical health. Thus, CME reduces gender differences in adult health, especially mental health.

**Table 8:** Effects of childhood migration distance on adult health

Variables	(1) Self-assessed health	(2) Physical health	(3) Mental health
Control group: no migration in childhood			
Cross-county migration	0.2086*** (0.0630)	0.2410*** (0.0677)	0.2309 (0.1485)
Cross-city migration	0.3376*** (0.1060)	0.0880 (0.1540)	0.4804* (0.2707)
Cross-province migration	0.1288 (0.1340)	0.0588 (0.1524)	0.1936 (0.3043)
Constant term		-1.0237*** (0.3227)	16.2609*** (0.6028)
Individual character	√	√	√
Family character	√	√	√
Region	√	√	√
N	7035	7035	7035
$$ {R}^{2}$$	0.0551	0.0216	0.0599

**Table 9:** Impact of childhood migration experience on adult health: gender differences

Variables	(1) Self-assessed health		(2) Physical health		(3) Mental health	
	Male	Female	Male	Female	Male	Female
migration	0.0783 (0.0781)	0.2805*** (0.0625)	0.2888*** (0.0921)	0.1387* (0.0743)	-0.2524 (0.1687)	0.5880*** (0.1508)
Constant term					16.4273*** (0.8499)	15.3962*** (0.9711)
Individual character	√	√	√	√	√	√
Family character	√	√	√	√	√	√
Region	√	√	√	√	√	√
SUEST	0.0657*		0.2082		0.0010***	
N	2942	4093	2942	4093	2942	4093
$$ {R}^{2}$$	0.0440	0.0528	0.0267	0.0252	0.0458	0.0512

## Conclusions and discussions

Existing studies have demonstrated that childhood experiences are associated with individual well-being across the lifespan, and this paper provides an essential explanation from the micro perspective of individuals. Based on data from the CLDS in 2016 and 2018, this paper explores the impact of CME on the adult health of rural migrants. The study found that CME can significantly improve rural migrants’ adult health, including physical health and mental health. Previous studies on China also confirm that rural migrant children have better physical and mental health compared to non-migrated rural children [42], and CME is positively associated with objective well-being [62]. Other studies in developed countries have shown that CME is more likely to negatively affect health [15–18]. Overall, the reasons underlying the health of childhood migrants may be complex, but the motivation to migrate is key, and the CME of rural migrants is a kind of “moving to opportunity”. Compared with urban local children, migrant children can get more family companionship, whose parents care about their life and study to the maximum extent, as laterally verified by many studies [4, 24, 62].

Migration distance and gender are also important factors affecting health. From the perspective of migration distance, in comparison with childhood non-migration, both cross-county and cross-city migration promoted the health status of rural migrants, but the positive effect of cross-province migration was not significant. Migration distance is an important economic factor as well as a geographic variable, and long-distance migration is more likely to damage the health of migrants [4, 63]. From the gender perspective, CME could more dramatically improve rural women’s adult health than men, especially in mental health. Some studies have shown that women are worse than men on most health indicators [46, 64]. Fortunately, the childhood migration of China’s rural population is more favorable to women, promoting health equality at the gender level.

Based on the above discussion, we draw the following insights: On the one hand, the CME of the rural migrants helps to improve adult health, and also reduces gender gaps in health, a positive effect that combines efficiency and equity, suggesting that it is crucial to promote the orderly migration of the rural residents to the city. With the “Citizenship of the Agricultural Transfer Population” policy, there will still be more rural migrants who migrate during their childhood. In this regard, the government should promote health strategies from a life-cycle perspective, intervene early in life, and strengthen the basic public service tied to the “permanent residents” to boost the family migration of rural children. On the other hand, attention can be focused on the long-distance migrating individuals, who should get more support through government intervention, community services and the intervention of social organizations, making the rural migrants better integrated into the cities. Besides, we should discard a stereotypical negative image of migrant children and their families. As Dong and Wang [65] pointed out in the book review, if we penetrate deeply into the life scenarios of migrant children, we would find that they are not as vulnerable as we think, experiencing more changes and forming a stronger ability to adapt to new environments.

The impact of migration is a popular theme in the field of development economics, and the findings of this paper provide a valid extension. First, unlike examining the impact of parental migration on children [66, 67], parental migration leads to the problem of left-behind children, so the “income effect” and the “care effect” are usually not balanced, resulting in rural children being potentially disadvantaged. Second, in contrast to the study of Lu et al. [47], which concluded that CME has a negative impact, but this paper uses the phrase “whether the place of residence at age 14 is the same as the place of birth” to imply the continuity of the migration time, and as far as possible excludes the situation of migration to the city and then back to the countryside again, avoiding estimation bias brought by returning to the countryside. Finally, we focus on the impact of CME on reducing gender differences in health, providing new insights into how gender shapes opportunities and access to resources across the life cycle.

Despite the extended work done in this paper, there still needs to be improved. First, because of data limitations, we cannot analyze the impact of age and times of childhood migrations; Second, the data used in this paper is retrospective, so respondents may have fuzzy memories which cause inaccurate information; third, CME is influenced by many factors, and the reasons for migration may be more important than the migration itself, so follow-up data that explore this would make the study more meaningful.

## Data Availability

No datasets were generated or analysed during the current study.
